# Fournier’s Gangrene Secondary to Acute Perforated Appendicitis

**DOI:** 10.7759/cureus.81905

**Published:** 2025-04-08

**Authors:** Adeoye Debo-Aina, Abigail Hardy, Alexander Martindale, Kayah Wilks, Rizwan Ahmed, Sonia Mason, Tarun Singhal, Nkwam Nkwam

**Affiliations:** 1 Urology, King’s College Hospital NHS Foundation Trust, London, GBR; 2 General and Colorectal Surgery, Princess Royal University Hospital, London, GBR; 3 Urology, Princess Royal University Hospital, King’s College Hospital NHS Foundation Trust, London, GBR; 4 General and Colorectal Surgery, Princess Royal University Hospital, King’s College Hospital NHS Foundation Trust, London, GBR; 5 General Surgery, Princess Royal University Hospital, London, GBR; 6 Anaesthesia, Princess Royal University Hospital, King’s College Hospital NHS Foundation Trust, London, GBR; 7 Colorectal Surgery, Princess Royal University Hospital, London, GBR

**Keywords:** atypical appendicitis, fournier gangrene, necrotising fasciitis, right inguinal hernia, right-sided hemicolectomy, debridement

## Abstract

Fournier’s gangrene (FG) is a life-threatening multi-aetiological and polymicrobial infective necrosis of the external genitalia, perineum, and peri-anal regions. Early recognition and aggressive multidisciplinary medical and surgical management are essential to reduce long-term morbidity and mortality. Acute appendicitis is a rare cause of FG. Here, we report the management of a 90-year-old man presenting atypically with a perforated appendicitis resulting in FG.

## Introduction

Fournier’s gangrene (FG) is a rare, synergetic polymicrobial necrotising soft tissue infection affecting the external genitalia, perineum, and perianal regions [1]. It predominantly affects males, with a high incidence of 10:1 in men [2], with 1.6 cases reported per 100,000 men often between the ages of 50 and 79 years [3]. FG is a life-threatening condition, with mortality as high as 40%-50% [4]. Risk factors associated with the development of FG include diabetes, obesity, renal disease, smoking, alcohol abuse, and immunosuppression [5].

Clinical presentation locally involves pain, erythema, oedema, skin necrosis, and crepitus. Systemically, it can present as pyrexia, sepsis, and shock [3]. The disease process is a rapidly progressing infective necrosis of fascia and subcutaneous tissue. Infection spreads along the fascial planes and is limited by the attachment of Colles’ fascia in the perineum, with involvement of the scrotum and penis, and extension to the anterior abdominal wall. If infective foci is urogenital, infection spread begins within Buck’s and Dartos’ fascia, and involve Colles’ fascia with limited involvement of the anal margin due to the attachment of Colles’ fascia to the perineal body. On the other hand, anorectal derived infections start within the perineum. Because of the intra-abdominal origin of the testicular vascular supply, which differs from that of the scrotum and perineum, the testes are often spared from the infective process [6].

FG can produce systemic complications, including single and multi-organ failure, besides the risks of mortality. Therefore, early recognition, with timely and aggressive surgical debridement and broad-spectrum antibiotic treatment via a multi-disciplinary approach, is important [7]. Acute appendicitis is a rare but identified cause of FG. This case study documents the presentation of FG in a 90-year-old patient with late-presenting appendicitis.

## Case presentation

A 90-year-old Caucasian male was brought to the emergency department by ambulance (day one) following a 24-hour history of antecedent scrotal/hernia pain. He had a medical history of a right inguinal hernia (that had recently increased in size with intermittent tenderness), benign prostatic hyperplasia (112 cc gland), hypertension, and hypercholesterolemia (ASA3), but remained independent with his activities of daily living and had a good functional baseline, mobilising with a stick. This progressed to a right scrotal swelling, with associated skin rupture and discharge of foul, offensive purulent content. On initial assessment, he denied abdominal pain and maintained a respiratory rate of 18 breaths/minute, with saturation of 96% on room air. His blood pressure was stable at 140/74 mmHg, with slight tachycardia at 102 beats/minute, while apyrexial (37°C). Initial laboratory investigations have been documented in Table 1. Initial resuscitation was commenced by the emergency department, with a single dose of amikacin, and amoxicillin and clavulanic acid (co-amoxiclav) were prescribed, along with a referral to urology for suspected FG.

**Table 1:** Laboratory results on admission.

Parameter (unit)	Result	Reference range
White cell count (×10^9^/L)	18.0	3.6–11.0
Haemoglobin (g/L)	123	130–180
Neutrophils (×10^9^/L)	16.7	1.8–7.5
Creatinine (μmol/L)	52	59–104
Estimated glomerular filtration rate (mL/minute)	89	>60
Na^+^ (mmol/L)	137	133–136
K^+^ (mmol/L)	5.6	3.5–5.3
C-reactive protein (mg/L)	125	<5
pH	7.40	7.35–7.45
pO_2_ (kPa)	10.2	11.0–13.0
pCO_2_ (kPa)	5.2	4.7–6.0
Lactate (mmol/L)	1.3	0.5–2.2

**Figure 1:**
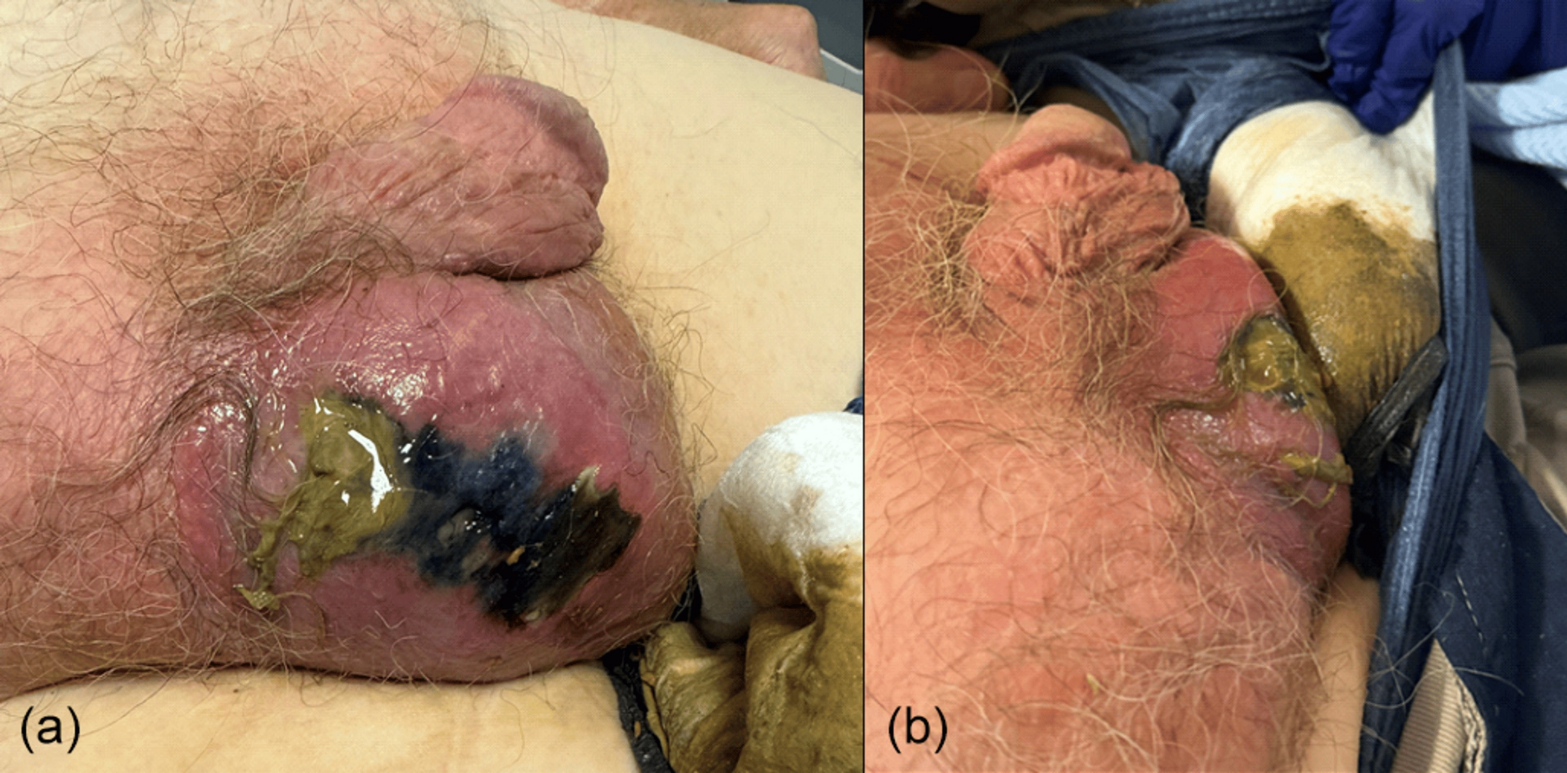
Erythematous right hemiscrotum with observed skin necrosis and purulent discharge at the site of skin break.

The patient was reviewed by the on-call urology team. He remained alert and haemodynamically stable. On examination, he had a soft, non-tender abdomen with an indurated right inguinal hernia that had a dusky appearance and was non-tender on palpation. Examination of external genitalia showed an enlarged right hemiscrotum, with an erythematous appearance of the skin with necrotic changes and malodorous discharge of pus (Figures 1a, 1b). There was no crepitus, or evidence of involvement of the penis, left hemiscrotum, or perineum. Examination findings were suggestive of FG. The patient was immediately consented for an emergency right scrotal exploration and debridement. A colorectal consult was also sought to exclude the possibility of a strangulated hernia, which was successfully excluded on reduction of his hernia and a soft, non-tender abdomen.

**Figure 2:**
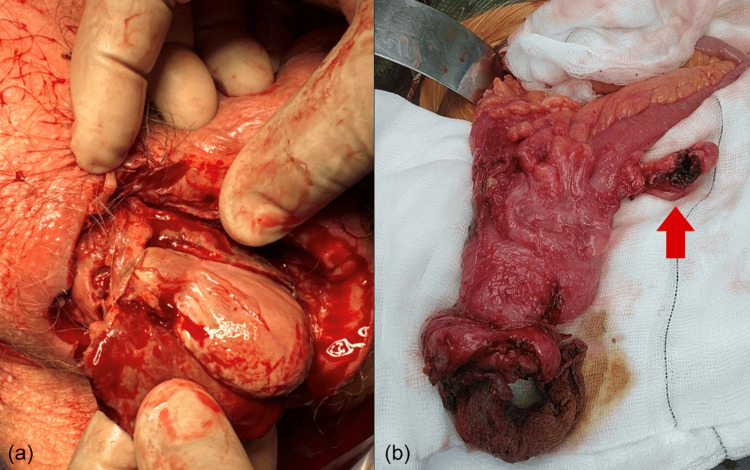
(a) Right testis: ruptured tunica vaginalis, with flaccid necrosed right testis. (b) Hernial sac content containing cecum and a perforated appendix (marked with an arrow).

The patient was catheterised with a 16 CH two-way silicon catheter. He underwent an expedient right scrotal exploration and debridement. Right hemiscrotal necrotic skin and tissue were excised and circumferentially debrided back to bleeding edges, extending up to the superficial ring. During exploration, the tunica vaginalis of the right testis was ruptured, revealing a flaccid and non-viable right testis (Figure 2a). As a result, he underwent a right scrotal orchidectomy. Debrided tissue specimens were collected for microscopy, culture, and sensitivities, along with the right testis for histological analysis. Following cord transfixation and excision of the right testis, faecal spillage was found extending from the inguinal canal. Assistance from the general surgical team was again sought in the operating room. He underwent a right groin exploration, with the hernial sac identified and the deep ring of the inguinal canal opened. On opening the hernial sac, contents included the cecum (which had been opened during scrotal exploration), as well as the ascending colon and part of the small bowel, pus collection, and a perforated appendix (Figure 2b). As such, an open right inguinal hernia repair as well as a laparotomy and right hemicolectomy with primary ileocolic anastomosis were performed. A Yeattes drain was inserted into the abdomen, and the midline wound was closed with skin clips.

**Figure 3:**
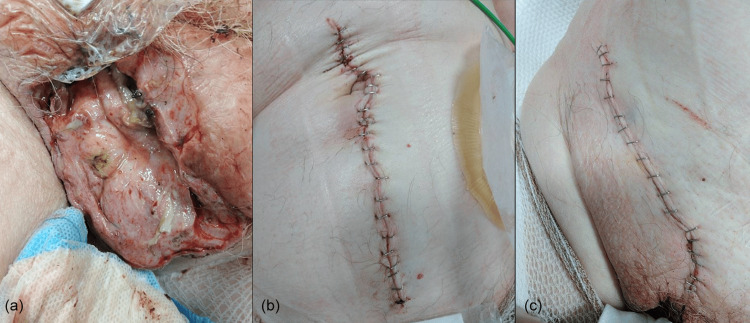
(a) Right hemiscrotum, post-surgical debridement and right orchidectomy. (b) Postoperative right hemicolectomy and primary anastomosis. (c) Right open inguinal hernia repair.

Post-procedure, the patient was transferred to the intensive care unit for continued inotropic support with metaraminol, wound review (Figures 3a-3c), and combined antibiotic treatment with amikacin, metronidazole, piperacillin, and tazobactam as per microbiology guidance. Patient was weaned off metaraminol and stepped down to ward-based care after 24 hours (day two).

**Figure 4:**
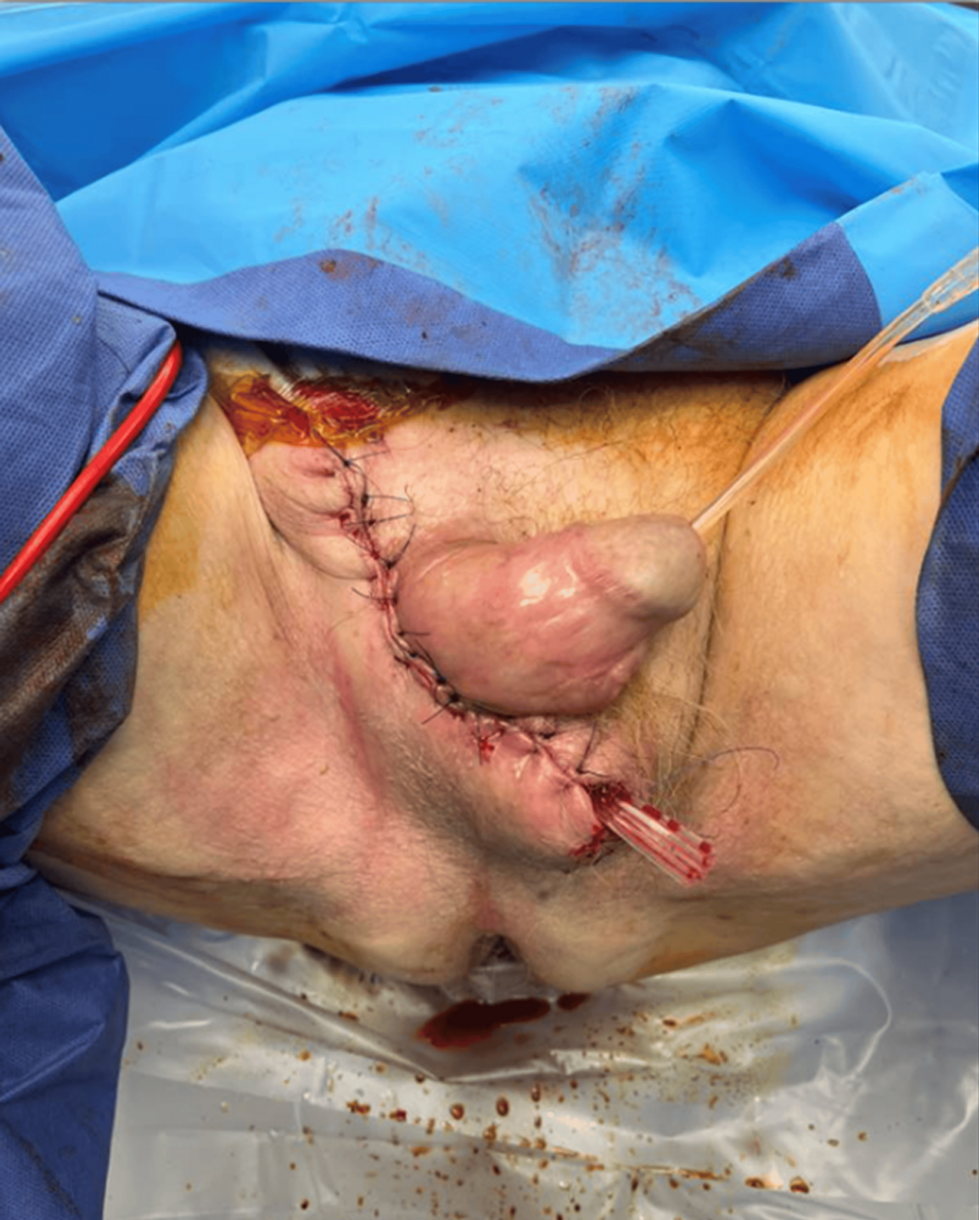
Postoperative relook debridement and wound closure.

On day three, the patient underwent a planned relook scrotal debridement. No significant new gangrenous processes were identified, with few areas of slough found along the wound and cavity margins which were debrided. After sufficient haemostasis, two-layer closure of scrotal wound was performed, with a Penrose corrugated drain left in situ (Figure 4).

**Figure 5:**
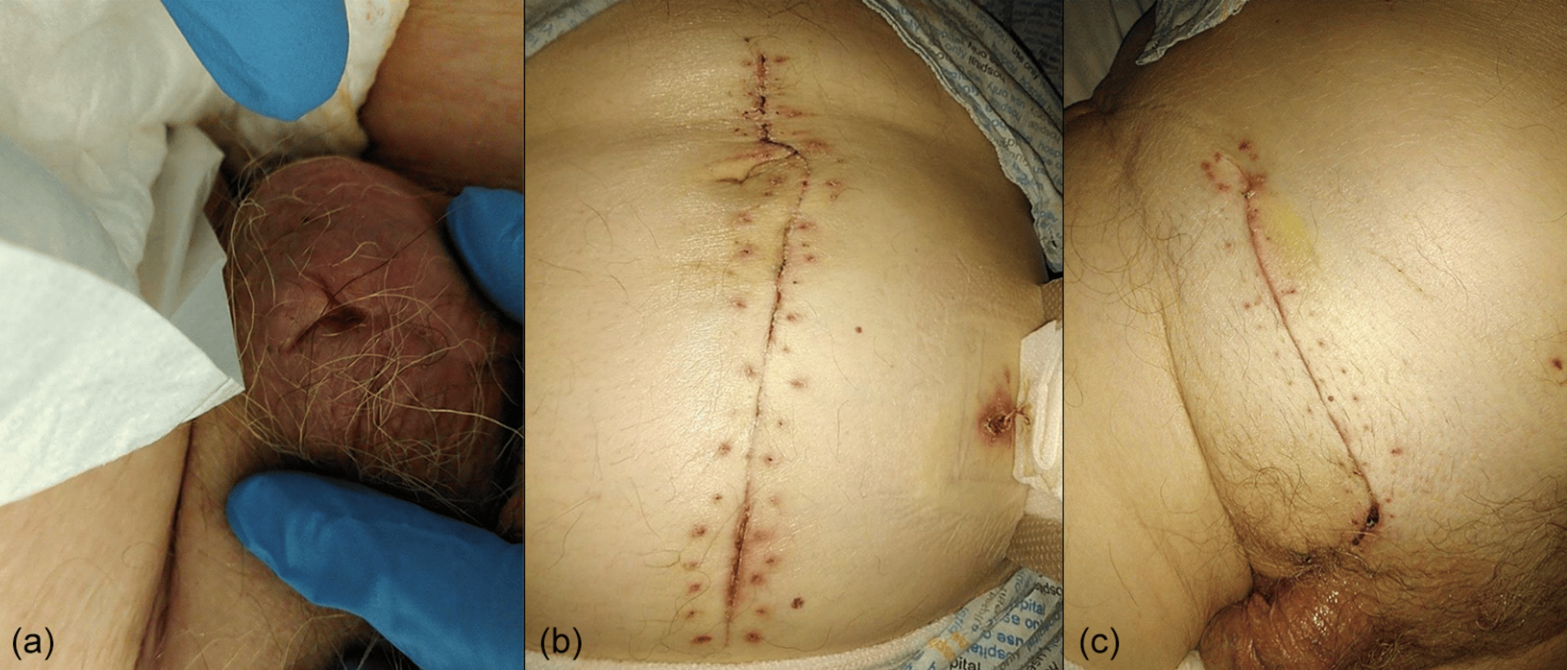
(a) Right hemiscrcotum following drain removal. Abdominal wound (b) and right inguinal hernia repair (c) following the removal of surgical clips.

The patient had continued ongoing ward-based management with regular wound reviews, physiotherapy, and slow escalation of diet. He had three episodes of vomiting and mild abdominal distension on day seven; however, this resolved without any intervention. First bowel motions occurred on day nine following initial right hemicolectomy and primary anastomosis after receiving laxatives. He then continued to regularly open his bowels. The scrotal drain was removed six days after his relook debridement (day nine), and the abdominal drain was removed on day 14 after the initial surgery. Alternate abdominal wound clips and scrotal sutures were removed on day 11. By day 14, all remaining abdominal wound clips and scrotal sutures were removed. He successfully had a trial without a catheter on day 15 and was made fit for discharge for further continuation of care at a bed-based rehabilitation unit. He was to continue on a soft, low-fibre diet for two weeks, with further outpatient follow-up arranged by the urology team and colorectal team at four weeks and six weeks, respectively (Figures 5a-5c).

Debrided scrotal tissue specimens collected for microbiological investigation, cultured heavily for *Escherichia coli*, *Streptococcus salivarius* (tissue samples), and anaerobes. These were sensitive to amikacin and penicillin. During the course of the patient’s admission, he completed seven days of intravenous piperacillin and tazobactam, metronidazole (six days), and two doses of amikacin. Additional antifungal cover was provided with fluconazole (six days). Histological analysis of the right testis demonstrated a gangrenous testis, epididymis, and spermatic cord with attached hernial sac. Microscopic assessment revealed evidence of gangrenous necrosis, acute and chronic inflammation, and no evidence of malignancy. Likewise, histological analysis of the resected cecum, appendix, and ileum revealed a gangrenous perforated appendix and partial thickness infarcts with mucosal ischemia in resected bowel segments, with no evidence of malignancy.

## Discussion

Acute appendicitis is a rare cause of FG. More so are the documented cases of FG arising secondary to appendicitis within a hernia, for which two other cases have been reported [8,9]. Gangrene often results from the perforated retrocecal or retroperitoneal positioned appendix with the subsequent dissemination of infection into the perineal and scrotal regions [10]. This is particularly the case when there is considerable pus and abscess formation near the deep inguinal ring [11]. Diagnosis of FG may be delayed if the underlying cause is intra-abdominal, resulting in a worse prognosis. This highlights the importance of excluding intra-abdominal infection in patients with FG, when the more common urinary tract, perirectal, and traumatic causes are not identified [12].

FG occurs because of aerobic and anaerobic bacterial infections involving the perineum and genitalia. The most common sites for infections are the ano-rectum (30-50%), uro-genitalia (20-40%), and genital surface [13]. Although infections are often polymicrobial (54%), involving both aerobic and anaerobic and gram-negative and gram-positive species. The most frequently isolated pathogen is *Escherichia coli* (46.6%), followed by *Streptococcus* (36.98%) [14]. Other associated pathogens include *Staphylococcus*, *Enterococcus*, *Pseudomonas*, *Enterobacter*, and *Klebsiella* [15]. In this case, cultures showed heavy growth of *Escherichia coli* and *Streptococcus*, confirming a polymicrobial infection.

FG can present asymptomatically in 40% of cases; however, most cases present clinically with features of scrotal or labial pain, erythema, cellulitis, abscesses, crepitus, and fever [16]. Our patient’s symptoms originally started with intermittent pain within the region of his hernia, which then progressed to right scrotal swelling; however, this did not alarm him to visit the hospital. Likewise, skin changes within the right hemiscrotum were not recognised until there was skin rupture and purulent malodorous discharge, late features of FG. Besides advanced age, at 90 years, our patient had no other identifiable risk factors for developing FG. Advanced age is associated with higher mortality with FG, as such clinical examination of the perineum, genitals, and abdomen for features of FG should be emphasised in older patients presenting with genital or perineal pain [14].

Radiological imaging serves a role in the diagnosis of FG in the form of computed tomography (CT). CT possess a higher specificity in FG diagnosis. It can lead to earlier diagnosis, accurate assessment of the extent of disease, and evaluation of perineal structures and retroperitoneum, where the disease can spread. Common CT findings are asymmetric fascial thickening, fluid collection, and abscess formation. The most defining feature of FG on CT is subcutaneous emphysema, which is not always present in all cases [17]. However, diagnosis is still clinical, as in our case, and initiation of treatment should be commenced urgently without delaying imaging.

Key study limitations include, first, reporting an atypical presentation of a perforated appendix within a hernia that has resulted in FG in an elderly individual. This atypical presentation is not a template for how other cases of FG secondary to appendicitis will present within the general and non-comorbid population groups. Furthermore, patient-specific factors such as the patient’s advanced age and underlying co-morbidities are likely to have influenced his outcome. Furthermore, this case report discusses only the immediate management of a single case of FG presenting secondary to appendiceal perforation, with no comparative analysis done with other published cases. Finally, although the patient had a positive outcome following the initial management, we do not have any follow-up data regarding his long-term outcome post-discharge. Hence, we are unable to comment on the potential long-term impact FG and its resultant management had on our patient.

## Conclusions

FG is a urological emergency with a high mortality rate, despite clinical and technological advancements in medical and surgical practice. Early recognition, commencement of resuscitative care, broad-spectrum antibiotics, and extensive surgical debridement of all devitalised tissue are essential in reducing mortality. This is well highlighted in our case, where prompt medical and surgical intervention reduced patient morbidity and mortality. A perforated appendicitis is a rare cause of FG; however, it is important to exclude the presence of appendicitis as a possible cause of FG, as seen in our case, because elderly and co-morbid patients may present with atypical symptoms of acute appendicitis.
